# Supplementary material for: Broadband multi-beam lens-assisted mmID enabling multi-gigabit backscatter data rates for next-generation wireless networks
Source: Nat Commun. 2026 Mar 10;17:3765. doi: 10.1038/s41467-026-70454-8 (PMC13106750; doi:10.1038/s41467-026-70454-8)
Supplement: Supplementary file 1 — Supplementary Information [file 41467_2026_70454_MOESM1_ESM.pdf]

Supplementary Information: Broadband Multi-Beam Lens-Assisted mmID Enabling Multi-Gigabit Backscatter Data Rates for Next Generation Wireless Networks

Marvin Joshi[1], Charles A. Lynch III[1], Kexin Hu[1], Yaw A. Mensah[1], John D. Cressler[1] and Manos M. Tentzeris[1]

[1]School of Electrical and Computer Engineering, Georgia Institute of Technology, Atlanta, GA 30332 USA

Supplementary Note 1: Backscatter Operation, Wideband Spectrum Measurement, and Backscatter Signal Modelling

This section provides a detailed description of the backscatter operation used in this work, together with a wideband spectrum measurement of the reflected signal and a mathematical model explaining the origin of the observed spectral components. Supplementary Figure 1 illustrates the frequency evolution of the waveform throughout the measurement chain. The process begins with a digitally generated baseband signal created by the arbitrary waveform generator. This waveform defines the desired modulation and occupies a narrowband region around baseband. It is then upconverted to a low-frequency subcarrier that drives the gate of the FET. In this work, a 2 GHz ASK excitation is used, producing two symmetric components at ± 2 GHz and modulating the transistor impedance in time.

The continuous-wave (CW) carrier generated by the reader is expressed as

$$x_{TX}(t) = A_{TX}\cos\big(2\pi f_c t + \theta_{TX}(t)\big),$$

where $x_{TX}(t)$ is the transmitted signal, A_{TX} is the carrier amplitude, f_c is the carrier frequency, and $\theta_{TX}(t)$ represents the phase noise of the source. For the measurements in this work, $f_c = 27$ GHz. At the mmID, the applied bias waveform drives the on-board FET, resulting in the modulated backscatter

$$x_{BS}(t) = A_{BS}\, m(t)\cos\big(2\pi(f_c + f_{IF})t + \theta_{TX}(t) + \phi\big),$$

where A_{BS} is the backscattered amplitude, $m(t)$ is the modulation waveform applied through the bias line, f_{IF} is the intermediate-frequency offset, and

$$\phi = -\frac{2\pi f_c R}{c}$$

is the one-way propagation phase delay. To explicitly show how sidebands arise in the reflected spectrum, we consider the single-tone modulation used in the measurement,

$$m(t) = \cos(2\pi f_{IF} t).$$

Substituting this into the backscatter expression produces a product of the carrier and modulation tones. Using the identity

$$\cos a \cos b = \tfrac{1}{2}\big[\cos(a+b) + \cos(a-b)\big],$$

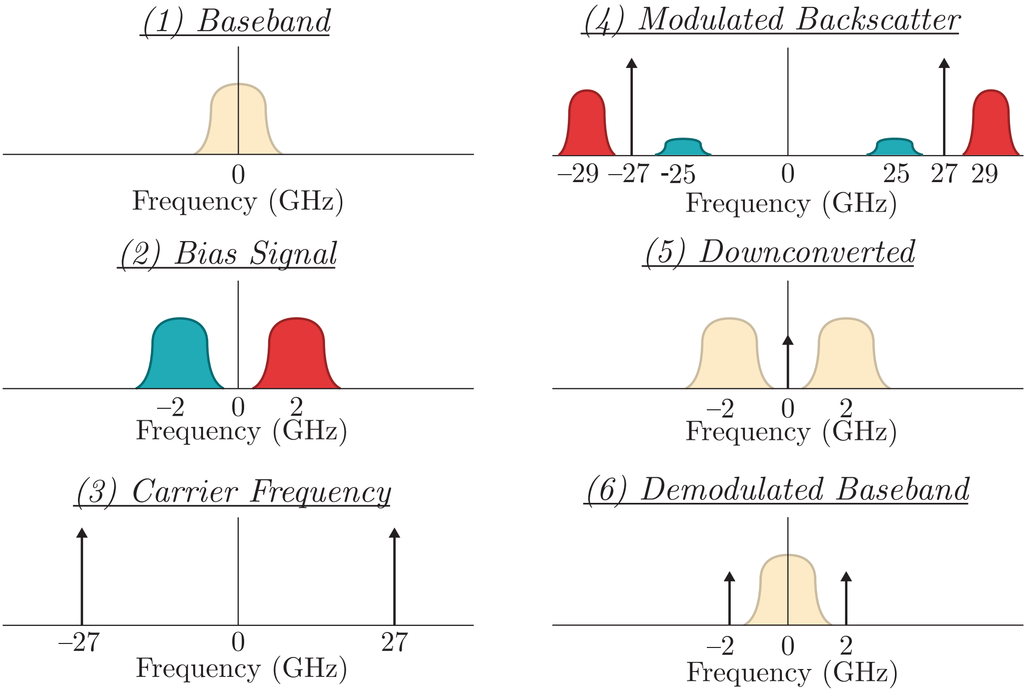

Supplementary Figure 1: Demonstration of the modulation and demodulation process used by the lens-based mmID to generate and return gigabit-per-second mmWave backscatter signals, including waveform generation, FET-based modulation, and reader-side downconversion.

the backscatter waveform expands to

$$x_{BS}(t) = \frac{A_{BS}}{2}\Big[\cos\big(2\pi(f_c + f_{IF})t + \theta_{TX}(t) + \phi\big) + \cos\big(2\pi(f_c - f_{IF})t + \theta_{TX}(t) + \phi\big)\Big].$$

This demonstrates that the reflected signal contains two frequency-translated tones:

$$f_{\mathrm{USB}} = f_c + f_{IF}, \qquad f_{\mathrm{LSB}} = f_c - f_{IF}.$$

With $f_c = 27\,\mathrm{GHz}$ and $f_{IF} = 2\,\mathrm{GHz}$, these reduce to

$$f_{\mathrm{USB}} = 29\,\mathrm{GHz}, \qquad f_{\mathrm{LSB}} = 25\,\mathrm{GHz}.$$

After free-space propagation, the received waveform at the reader is

$$x_{RX}(t) = \frac{A_{BS}}{R^{2n}}\, m(t)\cos\big(2\pi(f_c + f_{IF})t + \theta_{TX}(t) + 2\phi\big),$$

where R is the reader–tag separation and n is the path-loss exponent. The total round-trip phase shift is

$$2\phi = -\frac{4\pi f_c R}{c}.$$

Following IQ downconversion using a 27 GHz local oscillator, the recovered baseband signal becomes

$$x_{IQ}(t) = \frac{1}{2}A_{BS}m(t)\Big[\cos\big(\theta_{TX}(t) + \phi\big) + j\sin\big(\theta_{TX}(t) + 2\phi\big)\Big],$$

containing the in-phase (I) and quadrature (Q) components of the modulated backscatter.

To characterize the full spectral content of the reflected waveform, a wideband measurement of the backscatter was captured prior to any downconversion, as shown in Supplementary Figure 2. This spectrum correlates to *(4) Modulated Backscatter* step shown in Supplementary Figure 1. The dominant component corresponds to the upper sideband at 29 GHz, which lies within the strong-gain region of the antenna, dielectric lens, and receive chain. In contrast, the lower sideband at 25 GHz falls outside the high-gain region of this receive chain and therefore appears significantly attenuated, approaching the noise floor. All higher-order intermodulation components and harmonics similarly remain at or near background levels. The strongly frequency-selective response of the antenna, lens, and measurement receiver thus explains both the pronounced 29 GHz component and the suppressed out-of-band emissions observed across the measured spectrum.

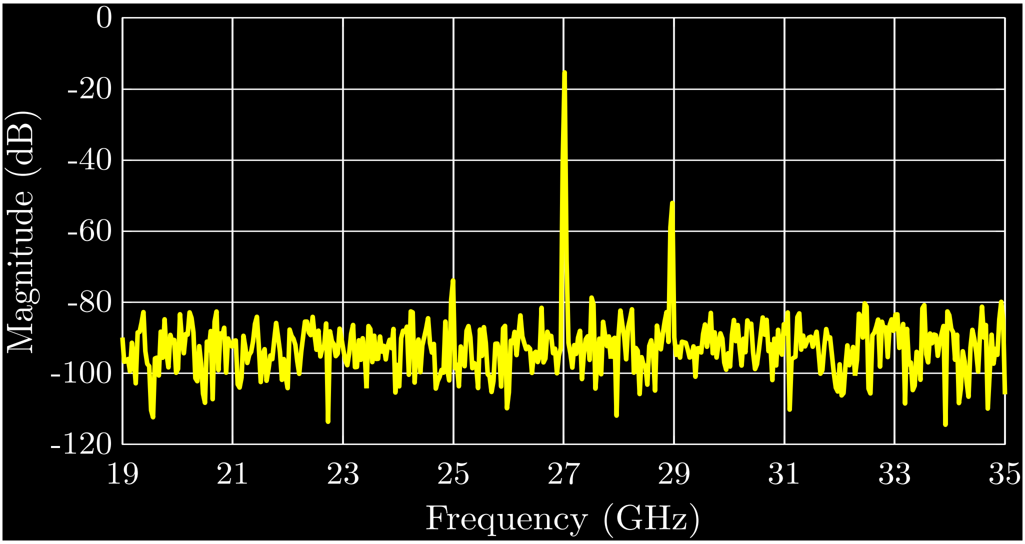

Supplementary Figure 2: Measured RF Spectrum of the mmWave Backscatter Signal Showing the Dominant Upper Sideband and Suppressed Lower Sideband

Supplementary Note 2: Time-Domain Verification of the Reflection-Mixing Mechanism

This section presents a time-domain verification of the reflection-based mixing operation in the proposed mmID tag. The measurement compares three waveforms: (a) the digitally generated 32 −QAM sequence produced by the arbitrary waveform generator, (b) the corresponding square-root raised-cosine (SRRC) shaped 2 GHz subcarrier that drives the FET gate, and (c) the received backscatter prior to any downconversion. These traces demonstrate that the backscattered signal retains the SRRC-shaped amplitude envelope imposed at the gate, enabling reliable recovery of the transmitted data.

The baseband 32 −QAM symbol sequence $s[k]$ is converted into a continuous-time waveform using SRRC pulse shaping,

$$x_{\mathrm{BB}}(t) = \sum_k s[k]\, p_{\mathrm{SRRC}}(t - kT_{\mathrm{sym}}),$$

where $p_{\mathrm{SRRC}}(t)$ is the normalized SRRC pulse and T_{sym} is the symbol period. The SRRC pulse occupies the two-sided bandwidth

$$W = \frac{1+\alpha}{T_{\mathrm{sym}}},$$

with roll-off factor $\alpha = 0.25$. This filtering produces the characteristic rounded symbol transitions visible in the time-domain envelope of the gate excitation. The pulse-shaped waveform is then upconverted to a 2 GHz subcarrier,

$$x_{\mathrm{sub}}(t) = x_{\mathrm{BB}}(t)\cos(2\pi f_{\mathrm{IF}} t),$$

and applied to the FET gate, where it modulates the reflection coefficient of the pixel at the 27 GHz carrier. The pixel is implemented in a cross-polarized configuration: the FET sits between two orthogonal polarization paths of the capacitive-coupled patch, so the reader receives only the time-varying component of the reflection coefficient. When the FET state is static, almost no energy couples into the receive polarization; modulation appears only when the reflection state varies in time. Because the modulation process is governed by multiplication of the 27 GHz CW field with the time-varying reflection coefficient, the relevant modulation depth is determined by the contrast between reflection states at the *carrier* frequency,

$$\Delta\Gamma = \left|\Gamma_1 - \Gamma_0\right|,$$

rather than by the behavior at the sideband frequencies. Figure 2(d) shows that even moderate gate voltages such as −0.25 V produce a sufficiently large $\Delta\Gamma$ at 27 GHz to generate the observed mixing products.

To verify this behavior experimentally, a direct time-domain measurement of the backscattered waveform was captured prior to any downconversion. Supplementary Figure 3 presents the baseband 32 −QAM sequence, the SRRC-shaped 2 GHz subcarrier applied to the FET gate, and the corresponding received backscatter. Although the backscatter is measured at very low received power and therefore exhibits visible noise, its amplitude envelope still follows the SRRC-shaped gate excitation. This confirms that the backscatter arises from the time-varying reflection coefficient at the 27 GHz carrier and that the modulation is preserved with sufficient fidelity to support high-order QAM demodulation.

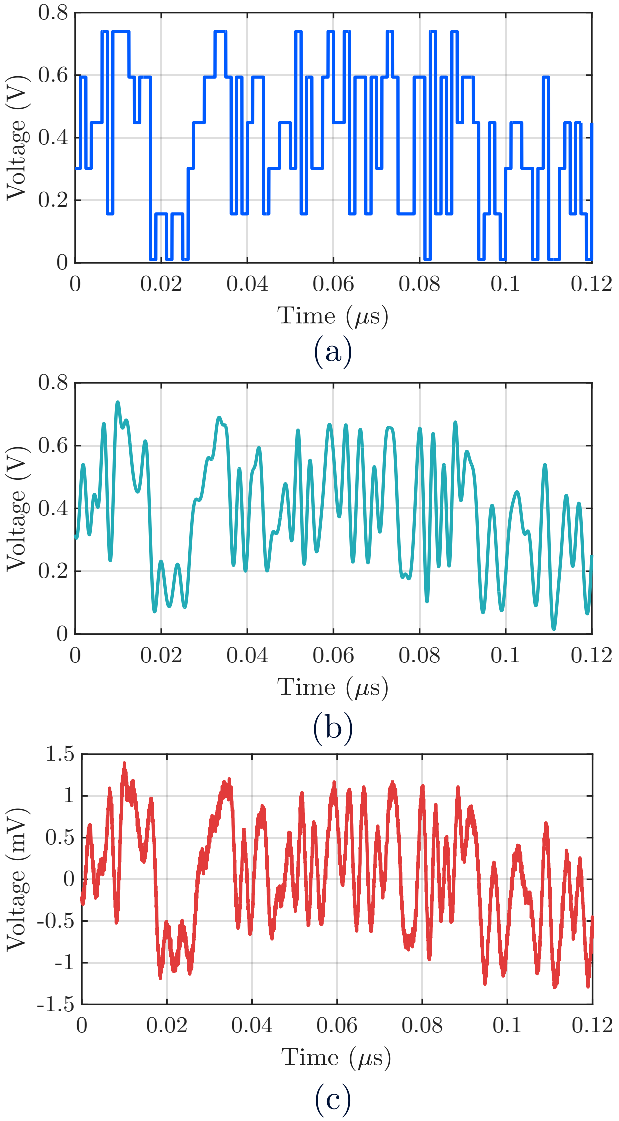

Supplementary Figure 3: Measured Time-domain signals used to verify reflection-based mixing: (a) Digitally generated $32-$QAM symbol sequence from the vector signal generator (VSG). (b) SRRC-shaped 2 GHz subcarrier waveform applied to the FET gate with roll-off factor $\alpha = 0.25$. (c) Measured backscatter signal from the mmID demonstrating that its amplitude envelope follows the SRRC-shaped gate excitation.
